# Lack of Associations of *CHRNA5-A3-B4* Genetic Variants with Smoking Cessation Treatment Outcomes in Caucasian Smokers despite Associations with Baseline Smoking

**DOI:** 10.1371/journal.pone.0128109

**Published:** 2015-05-26

**Authors:** Rachel F. Tyndale, Andy Z. X. Zhu, Tony P. George, Paul Cinciripini, Larry W. Hawk, Robert A. Schnoll, Gary E. Swan, Neal L. Benowitz, Daniel F. Heitjan, Caryn Lerman, PGRN-PNAT Research Group

**Affiliations:** 1 Campbell Family Mental Health Research Institute, Centre for Addiction and Mental Health (CAMH), Toronto, Ontario, Canada; 2 Department of Pharmacology and Toxicology, University of Toronto, Toronto, Ontario, Canada; 3 Division of Brain & Therapeutics, Department of Psychiatry, University of Toronto, Toronto, Ontario, Canada; 4 Department of Behavioral Science, University of Texas MD Anderson Cancer Center, Houston, Texas, United States of America; 5 Department of Psychology, University at Buffalo, State University of New York (SUNY), Buffalo, New York, United States of America; 6 Center for Children and Families, University at Buffalo, State University of New York (SUNY), Buffalo, New York, United States of America; 7 Center for Interdisciplinary Research on Nicotine Addiction, Department of Psychiatry, University of Pennsylvania, Philadelphia, Pennsylvania, United States of America; 8 Department of Medicine, Stanford University, Palo Alto, California, United States of America; 9 Department of Medicine, University of California San Francisco, San Francisco, California, United States of America; 10 Department of Bioengineering & Therapeutic Sciences, University of California San Francisco, San Francisco, California, United States of America; 11 Department of Biostatistics & Epidemiology, University of Pennsylvania, Philadelphia, Pennsylvania, United States of America; Duke Cancer Institute, UNITED STATES

## Abstract

*CHRNA5-A3-B4* variants, rs16969968, rs588765 and rs578776, are consistently associated with tobacco consumption among smokers, but the association with smoking cessation is less consistent. Among the studies that reported significant associations with cessation, the effects were observed in smokers treated with placebo treatment in some studies and conversely in those receiving active pharmacological therapy (bupropion and nicotine replacement therapies) in others. Thus, it remains unclear whether *CHRNA5-A3-B4* is a useful marker for optimizing smoking cessation. Using data from 654 Caucasian smokers treated with placebo, nicotine patch or varenicline, we investigated whether *CHRNA5-A3-B4* variants were associated with smoking cessation outcomes, and whether there were significant genotype-by-treatment or haplotype-by-treatment interactions. We observed no significant associations between *CHRNA5-A3-B4* variants and smoking cessation, despite replicating previous associations with baseline tobacco consumption. At end of treatment the effect size on smoking cessation in the placebo, patch and varenicline groups for rs16969968 [GG vs. GA+AA] was OR = 0.66 (P = 0.23), OR = 1.01 (P = 0.99), and OR = 1.30 (P = 0.36) respectively, of rs588765 [CC vs. CT+TT] was OR = 0.96 (P = 0.90), OR = 0.84 (P = 0.58), and OR = 0.74 (P = 0.29) respectively, and for rs578776 [GG vs. GA+AA] on smoking cessation was OR = 1.02 (P = 0.95), OR = 0.75 (P = 0.35), and OR = 1.20 (P = 0.51) respectively. Furthermore, we observed no associations with cessation using the *CHRNA5-A3-B4* haplotype (constructed using rs16969968 and rs588765), nor did we observe any significant genotype-by-treatment interactions, with or without adjusting for the rate of nicotine metabolism (all P>0.05). We also observed no significant genetic associations with 6 month or 12 month smoking abstinence. In conclusion, we found no association between *CHRNA5-A3-B4* variants and smoking cessation rates in this clinical trial; however, as expected, significant associations with baseline tobacco consumption were replicated. Our data suggest that *CHRNA5-A3-B4* gene variants do not exhibit a robust association with smoking cessation and are unlikely to be useful for clinically optimizing smoking cessation pharmacotherapy for Caucasian smokers.

## Introduction

Smoking is a leading cause of premature death; world-wide about 6 million deaths each year can be attributed to smoking [1]. Compared to never smokers, smokers’ life expectancy is reduced by an average of 10 years [2]. Nicotine is the main psychoactive component of tobacco and exerts its pharmacological effects by its actions on the nicotinic acetylcholine receptors [3]. Genetic variants in *CHRNA5-A3-B4*, encoding the α5, α3, and β4 nicotinic acetylcholine receptor subunits, are robustly associated with smoking behaviors such as cigarettes smoked per day and nicotine dependence, as well as risks for smoking-related diseases, including lung cancer and chronic obstructive pulmonary disease [4–10]. Genetic mapping studies indicate that three independent loci within *CHRNA5-A3-B4* are associated with cigarette consumption and nicotine dependence in Caucasians [11, 12]. These independent loci can be represented by rs16969968 and correlated SNPs (sometimes referred to as “Bin A” or “Locus 1”), rs588765 and correlated SNPs (sometimes referred to as “Bin B” or “Locus 3”), and rs578776 and correlated SNPs (sometimes referred to as “Bin C” or “Locus 2”). The impact of these independent loci on cigarette consumption and nicotine dependence has been consistently replicated, but whether these variants also predict smoking cessation outcomes is less clear and is the focus of this investigation [11, 12].

Smoking cessation at any age has tremendous health benefits. Smokers who had quit smoking at 30, 40 and 50 years of age gained an average of 10, 9, and 6 years of life, respectively, when compared with those who continued to smoke [2]. Yet despite the substantial health benefits, only 3% of all smokers are able to quit smoking each year [3]. Of the three FDA-approved smoking cessation treatments [1], transdermal nicotine patch delivers nicotine to reduce craving and withdrawal in smokers to promote smoking cessation. It is a commonly used treatment with few side effects, but it has modest clinical efficacy. Varenicline, a partial agonist for the α4β2 nicotinic receptor and a full agonist for the α7 nicotinic receptor [13], appears to have the greatest clinical efficacy, but it has more side effects, such as nausea, which can lead to discontinuation of use. Substantial individual variability is observed in both clinical efficacy and in side effects within each type of smoking cessation treatment; genetics could contribute to this variability [14–16]. The estimated heritability of smoking cessation is 50–58% indicating that genetic factors are important determinants of cessation [14, 17]. Genetically tailored drug therapy could assist in maximizing smoking cessation efficacy. For example, the Pharmacogenomics of Nicotine Dependence Treatment (PNAT) clinical trial demonstrated that varenicline was more efficacious than nicotine patch for smokers with genetically normal rates of nicotine metabolism, while the efficacy was equivalent for smokers with genetically slower rates of nicotine metabolism. Smokers with slower rates of nicotine metabolism (vs. smokers with normal rate of nicotine metabolism) also had higher overall side-effect severity when taking varenicline in comparison to placebo; together this suggests that normal metabolizers should be treated with varenicline, while slow metabolizers should be treated with nicotine patch [18].

A number of studies have investigated the association between *CHRNA5-A3-B4* gene variants and smoking cessation with inconsistent results. Some studies observed significant associations between *CHRNA5-A3-B4* gene variants and smoking cessation [19–23], while other studies did not [24–29], and a large genome-wide association study did not find a significant association between *CHRNA5-A3-B4* gene variants and smoking status (i.e. current versus former smokers) [5]. Even among those studies reporting a significant association between *CHRNA5-A3-B4* gene variants and smoking cessation, it is not clear whether the *CHRNA5-A3-B4* SNPs have a general effect on smoking cessation, which is independent of the specific type of pharmacological treatment (i.e., would alter all active treatments), or whether they alter the efficacy of a specific treatment arm (i.e. only nicotine patch or placebo). For example, Chen and colleagues reported that the association between a *CHRNA5-A3-B4* haplotype, the rs16969968_rs588765, consisting of rs16969968 (Bin A) and rs680244/rs588765 (Bin B), and smoking cessation was primarily observed among smokers treated with placebo; and that this association was mitigated by active pharmacological therapy [30]. In contrast, Munafò and colleagues reported a significant association between *CHRNA5-A3-B4* variant rs1051730 (Bin A) and smoking cessation outcomes in smokers who were receiving nicotine replacement therapy, with little effect observed in those receiving placebo treatment [21]. Another recent study reported a significant treatment (placebo vs. nicotine replacement therapy) by genotype (rs1051730) interaction on smoking cessation outcomes [31].

The objective of this study was to investigate the association between *CHRNA5-A3-B4* gene variants and the clinical efficacy (i.e. smoking cessation outcomes) associated with treatment with placebo, nicotine patch or varenicline. This pharmacogenomics trial is the first to compare the impact of *CHRNA5-A3-B4* gene variants on smoking cessation outcomes in these two widely used treatments head-to-head while including a double-dummy placebo controlled design. Further, the placebo treatment arm (N = 212) is larger than that included in the prior report identifying significant associations with *CHRNA5-A3-B4* gene variants and smoking cessation under placebo treatment (N = 132)[30]. If these SNPs are validated in the current trial as significantly associated with an active treatment, it could be possible to optimize the choice of pharmacotherapy for individual smokers based on genotype or haplotype. We first sought to confirm the significant effect of the *CHRNA5-A3-B4* variants on baseline smoking behaviors in the screened Caucasian population (N = 1026), and then to investigate the association between these *CHRNA5-A3-B4* SNPs and smoking cessation in 654 Caucasian smokers randomized to placebo, nicotine patch or varenicline. We focused on the Caucasians participants of the clinical trial in this analysis to minimize population stratification.

## Methods

### Participants and procedures

Smokers were recruited into a placebo-controlled clinical trial through advertisements for a free smoking cessation program. The primary findings of this clinical trial were recently published [18]. The study consisted of three treatment arms: (1) nicotine patch (placebo pill/active patch); (2) varenicline (active pill/placebo patch); and (3) placebo (placebo pill/placebo patch). The present genetic analyses were restricted to Caucasian smokers to minimize population stratification as the frequency of these variants varies substantially between world populations. Caucasian smokers (N = 1026) were screened for their rate of nicotine metabolism (based on their nicotine metabolite ratio) and baseline smoking phenotypes. Of this group, 654 Caucasian smokers were randomized into the three treatment arms with slow metabolizers oversampled to assess the influence of nicotine metabolism on smoking cessation [18]. The over-sampling of smokers with slow nicotine metabolism is unlikely to introduce bias into our analyses of *CHRNA5-A3-B4* and smoking cessation since the allele frequencies of the *CHRNA5-A3-B4* SNPs observed in this study were very similar to allele frequencies reported by previous studies in Caucasians as well as by the 1000 genome project (see results) [11, 12, 30]. Furthermore, the gene encoding for the main nicotine metabolizing enzyme, CYP2A6, is on a different chromosome than *CHRNA5-A3-B4*, and is thus unlikely to have any linkage equilibrium relationship with *CHRNA5-A3-B4*. There was also little difference in the results with or without adjusting for the rate of nicotine metabolism in any of the smoking cessation models.

The clinical trial was conducted at four academic medical centers (University of Pennsylvania, Centre for Addiction and Mental Health/University of Toronto, State University of New York at Buffalo, and MD Anderson Cancer Center). Eligible participants were 18–65 years old and reported smoking ≥ 10 cigarettes per day for ≥ 6 months (verified by carbon monoxide (CO) > 10ppm). Exclusion criteria included use of non-cigarette tobacco products; history of substance abuse treatment (other than nicotine); consumption of more than 25 standard alcohol drinks per week; history of DSM-IV Axis 1 psychiatric disorder or suicide risk score on the MINI International Neuropsychiatric Interview; history of cancer, kidney or liver diseases; history of clinically significant cardiac dysrhythmias, stroke, angina, heart attack, or uncontrolled hypertension; use of smoking cessation medication within the last 14 days; current use of anti-psychotics, stimulants, opiate medications, anti-coagulants, rescue inhalers, or antiarrhythmics; and pregnancy/lactation. The study protocol has been published previously [18]. Briefly, intention-to-treat smokers were randomly assigned by baseline NMR status to 11 weeks of placebo (placebo pill plus placebo patch), 21 mg nicotine patch (active patch plus placebo pill), or 1 mg varenicline (active pill plus placebo patch), plus behavioral counselling. The primary endpoint was biochemically verified 7 day point prevalence abstinence at the end of treatment to estimate the pharmacological effect of treatment by NMR. All participants provided written informed consent in accordance with the principles expressed in the Declaration of Helsinki, and the study protocol was approved by the institutional review boards of University of Pennsylvania, University of Toronto, State University of New York at Buffalo, and MD Anderson Cancer Center.

### Smoking abstinence

Biochemically verified 7-day point prevalence abstinence was assessed at end of treatment (EOT, week 11), 6 months and 12 months. Point prevalence abstinence is defined as no-self-reported smoking (not even a puff) for at least 7 days and was verified by exhaled carbon monoxide levels < 8 ppm [32]. We focused on the EOT time point in the present study as we were interested in the pharmacogenetic effect of *CHRNA5-A3-B4* variants on smoking cessation, which is strongest during treatment; as in most pharmacogenomics studies this was the primary time point used for analysis of these gene variants [21, 23, 30, 31]; we have also included 6 month and 12 month smoking abstinence association data in supplementary materials. All statistical analyses on smoking cessation were performed on an intent-to-treat basis; subjects lost during follow-up were considered as smokers [32].

### Genotyping

A total of 1026 DNA samples were extracted from blood. The *CHRNA5-A3-B4* SNPs were genotyped using ABI Viia 7 real time PCR machine (Applied Biosystems, Foster City, CA). The genotyping reaction was performed with 2.5 μL TaqMan GTXpress master mix and 2.5 μL of water containing 10 ng of DNA and 0.0625 μL of 80x Taqman SNP genotyping probes (rs16969968: C__26000428_20, rs588765: C____18826_10 and rs578776: C____721253_10, Applied Biosystems, Foster City, CA). The allele discrimination data were analyzed using Viia 7 software version 1.2. All genotyping results included in our analyses had call quality values >0.985. For quality control (QC) all samples were genotyped again using a second independent DNA sample for two high allele frequency variants (rs16969968 & rs588768), and ~18% of the samples (n = 180) were also re-genotyped for rs578776. The concordance rates for all SNPs between the original genotyping and the QC genotyping were 100%.

PLINK was used to estimate *CHRNA5-A3-B4* haplotype (rs16969968 and rs588765) probabilities for each individual (using the—hap-phase-wide option) as described previously [30, 33]. The posterior probability for the haplotypes was 1 for more than 99% of the haplotypes. The haplotypes were assumed to be ‘additive’ [30].

### Nicotine metabolism

This clinical trial was design to prospectively assess the impact of nicotine metabolism on smoking cessation outcomes. The rate of nicotine metabolism was assessed in a baseline blood sample by the nicotine metabolite ratio (NMR; 3’hydroxycotinine/cotinine). NMR is highly reproducible in regular smokers, shows little temporal fluctuation due to the long half-life of cotinine (16 hours) [34, 35], and is not influenced by variation in other cotinine or 3-hydroxycotinine metabolizing genes, but rather exclusively by CYP2A6 [36]. The NMR used to distinguish slow metabolizers from normal metabolizers was 0.31 based on the stratification performed in the parent clinical trial [18]. Individuals with slower rates of nicotine metabolism were over-sampled to increase statistical power, therefore, in the current analyses we performed analyses with and without adjusting for the rate of nicotine metabolism (as a covariate) in all the smoking cessation models. Thus these models examined both the role of the nicotinic receptor variants alone, and with the CYP2A6 phenotype as previously described [37]. Additional baseline measures assessed included self-report cigarettes per day, cotinine levels, derived cotinine per cigarette and Fagerstrom test for nicotine dependence (FTND).

### Analytical chemistry

Concentrations of cotinine and 3-HC were determined by liquid chromatography-tandem mass spectrometry (LC-MS/MS) as described previously [38, 39]. Briefly, 100 μL biological samples were extracted using a liquid-liquid extraction with cotinine-d_3_ and 3-hydroxycotinine-d_3_ as the internal standards. The evaporated extract was reconstituted in 100 μL of 100 mM aqueous ammonium acetate: methanol (v/v 80:20) with 1% acetic acid [38, 39]. The chromatography was carried out using a Synergi Polar RP column (150 x 4.6 mm I.D.; particle size 4 micron). A linear gradient of 10 mM ammonium acetate/0.1% acetic acid in water (solvent A) and 10 mM ammonium acetate/0.1% acetic acid in methanol (solvent B) was used as the mobile phase [38, 39]. At a flow rate of 0.7 ml/min. 3-HC and cotinine eluted at 5.3 and 6.2 min, respectively. Calibration standards were prepared in the range of 1 to 1000 ng/mL, unlabeled standards were plotted against fixed concentration (20 ng/ml) of corresponding labeled internal standard. The plots were linear for cotinine and 3HC with r^2^ > 0.99 for both. Cotinine’s molecular weight is 176.21g/mol. Thus, 1 ng/ml cotinine = 5.67 nmol/L.

### Statistical analyses

Baseline demographic variables among the total screened population were compared between *CHRNA5-A3-B4* genotypes using Mann-Whitney tests, Kruskal–Wallis tests, or Chi-square tests. The associations between dichotomized *CHRNA5-A3-B4* SNPs rs16969968 (G/G = 0 and G/A and A/A = 1), rs588765 (C/C = 0 and C/T and T/T = 1), and rs578776 (A/A = 0 and A/G and G/G = 1) and smoking abstinence were evaluated using logistic regression among the intent to treat population. All smoking cessation models were adjusted for age, gender and rate of nicotine metabolism as covariates (see figure legends for details). The *CHRNA5-A3-B4* haplotypes were dummy coded (i.e., each individual would have variables for haplotype 1, haplotype 2 and haplotype 3). Statistical analyses were performed using Stata 12 (StataCorp, College Station, TX).

## Results

### Descriptive data of the study participants

Demographic data for the screened population are presented in Table 1. The study participants smoked on average 20.2 cigarettes per day, and 43% of the study participants were female. Smokers with a slower rate of nicotine metabolism (i.e., NMR<0.31) smoked 19.1 cigarettes per day (95% CI = 18.4–19.8), whereas smokers with a normal rate of nicotine metabolism smoked 20.6 cigarettes per day (95% CI = 20.0–21.2, P = 0.004). All three genotyped *CHRNA5-A3-B4* SNPs were in Hardy-Weinberg equilibrium (all P>0.05, Table 1). The SNPs rs16969968, rs588765 and rs578776 had minor allele frequencies of 39.3%, 40.0% and 25.0% respectively in the screened population (N = 1026), which were very similar to 37.3%, 41.7% and 25.2% in the intent-to-treat participants (N = 654). These allele frequencies were also very similar to published frequencies in Caucasians (e.g. Saccone et al., 2010 reported 42%, 39% and 24% respectively [12], and the 1000 genome project reported 41.7%, 39.2% and 24.2% respectively) [4, 5, 11, 12, 30, 40]. *CHRNA5-A3-B4* haplotypes were constructed using rs16969968 and rs588765 [30]. In this study, the rs16969968_rs588765 haplotype frequencies were G_C 20.9%, G_T 40.0%, and A_C 39.2%, which is very similar to the 20.8%, 43.7% and 35.5% reported previously [30]. Those were labeled haplotype 1, haplotype 2, and haplotype 3 respectively [30].

**Table 1:** Baseline demographics and smoking behaviors in the screened population (N = 1026).

**RS16969968**				
	**GG**	**GA**	**AA**	P
*n* (screened)^a^	370	506	150	
Cigarettes per day	19.8 (18.9–20.7)	20.2 (19.6–20.9)	21.0 (19.2–22.2)	0.30
CO levels *only ITT	23.5 (22.2–24.8)	25.8 (24.5–27.0)	24.9 (22.4–27.3)	0.05
Cotinine (ng/mL)	219.6 (209.4–229.8)	242.0 (232.3–251.6)	269.0 (252.5–285.4)	**<0.0001**
3'-Hydroxycotinine (ng/mL)	91.0 (84.9–97.1)	93.7 (88.9–98.5)	116.4 (104.3–128.5)	**<0.0001**
Sum of Cotinine and 3'-Hydroxycotinine (ng/mL)	311.1 (296.3–325.8)	336.1 (322.8–349.3)	385.3 (359.7–411.0)	**<0.0001**
FTND	5.2 (5.0–5.4)	5.4 (5.1–5.8)	5.4 (5.1–5.8)	0.23
NMR	0.42 (0.40–0.45)	0.40 (0.39–0.42)	0.43 (0.40–0.47)	0.21
Sex (% Female)	41%	44%	42%	0.67
Age (Years)	46.3 (45.2–47.5)	46.1 (45.1–47.1)	44.8 (42.8–46.7)	0.33
BMI (Kg/m2)	29.1 (28.5–29.7)	28.4 (27.9–28.9)	29.5 (28.4–30.6)	0.08
**RS588765**				
	**CC**	**CT**	**TT**	P
*n* (screened)^b^	360	511	155	
Cigarettes per day	20.5 (19.6–21.2)	20.2 (19.4–20.9)	19.6 (18.6–20.6)	0.56
CO levels *only ITT	25.4 (23.9–26.9)	24.7 (23.5–25.8)	24.0 (21.9–26.1)	0.52
Cotinine (ng/mL)	243.8 (232.9–254.6)	238.7 (229.2–248.2)	220.7 (204.9–236.6)	0.07
3'-Hydroxycotinine (ng/mL)	99.7 (93.2–106.3)	96.2 (90.9–101.4)	86.9 (78.7–95.2)	0.09
Sum of Cotinine and 3'-Hydroxycotinine (ng/mL)	344.2 (328.4–360.1)	335.3 (321.9–348.7)	307.7 (285.6–329.7)	**0.04**
FTND	5.4 (5.1–5.6)	5.3 (5.1–5.5)	5.3 (5.0–5.6)	0.87
NMR	0.41 (0.39–0.43)	0.42 (0.40–0.44)	0.41 (0.37–0.44)	0.81
Sex (% Female)	40%	46%	38%	0.91
Age (Years)	46.3 (45.1–47.4)	46.2 (45.2–47.2)	44.5 (42.8–46.3)	0.22
BMI (Kg/m2)	29.0 (28.4–29.6)	28.6 (28.1–29.1)	29.2 (28.2–30.1)	0.41
**RS578776**				
	**GG**	**GA**	**AA**	P
*n* (screened)^c^	572	396	58	
Cigarettes per day	20.3 (19.7–20.9)	19.9 (19.1–20.7)	20.7 (18.4–23.1)	0.61
CO levels *only ITT	24.8 (23.6–25.9)	24.8 (23.5–26.1)	25.1 (20.3–29.8)	0.99
Cotinine (ng/mL)	246.4 (237.3–255.4)	230.9 (220.9–40.9)	200.7 (176.3–224.2)	**0.002**
3'-Hydroxycotinine (ng/mL)	99.6 (94.6–104.7)	91.9 (86.0–97.9)	88.5 (75.2–101.9)	0.09
Sum of Cotinine and 3'-Hydroxycotinine (ng/mL)	346.0 (333.1–358.9)	323.8 (309.5–338.2)	288.8 (254.7–322.9)	**0.005**
FTND	5.4 (5.2–5.5)	5.3 (5.1–5.4)	5.4 (4.8–6.0)	0.68
NMR	0.42 (0.40–0.43)	0.40 (0.39–0.43)	0.46 (0.40–0.52)	0.16
Sex (% Female)	44%	42%	38%	0.70
Age (Years)	45.0 (44.1–46.0)	47.1 (46.1–48.2)	47.3 (44.4–50.2)	**0.01**
BMI (Kg/m2)	28.8 (28.2–29.2)	29.0 (28.5–29.6)	28.1 (26.8–29.5)	0.51

^a^ RS16969968: there were 250, 320 and 84 intent-to-treat smokers in the GG, GA and AA group respectively.

^b^ RS588765: there were 219, 325, 110 intent-to-treat smokers in the CC, CT and TT group respectively.

^c^ RS578776: there were 359, 260, 35 intent-to-treat smokers in the GG, GA and AA group respectively.

NMR = nicotine metabolite ratio. FTND = Fagerstrom Test for Nicotine Dependence.

### Association between *CHRNA5-A3-B4* genetic variants and cigarettes consumption

#### rs16969968

**Fig 1:**
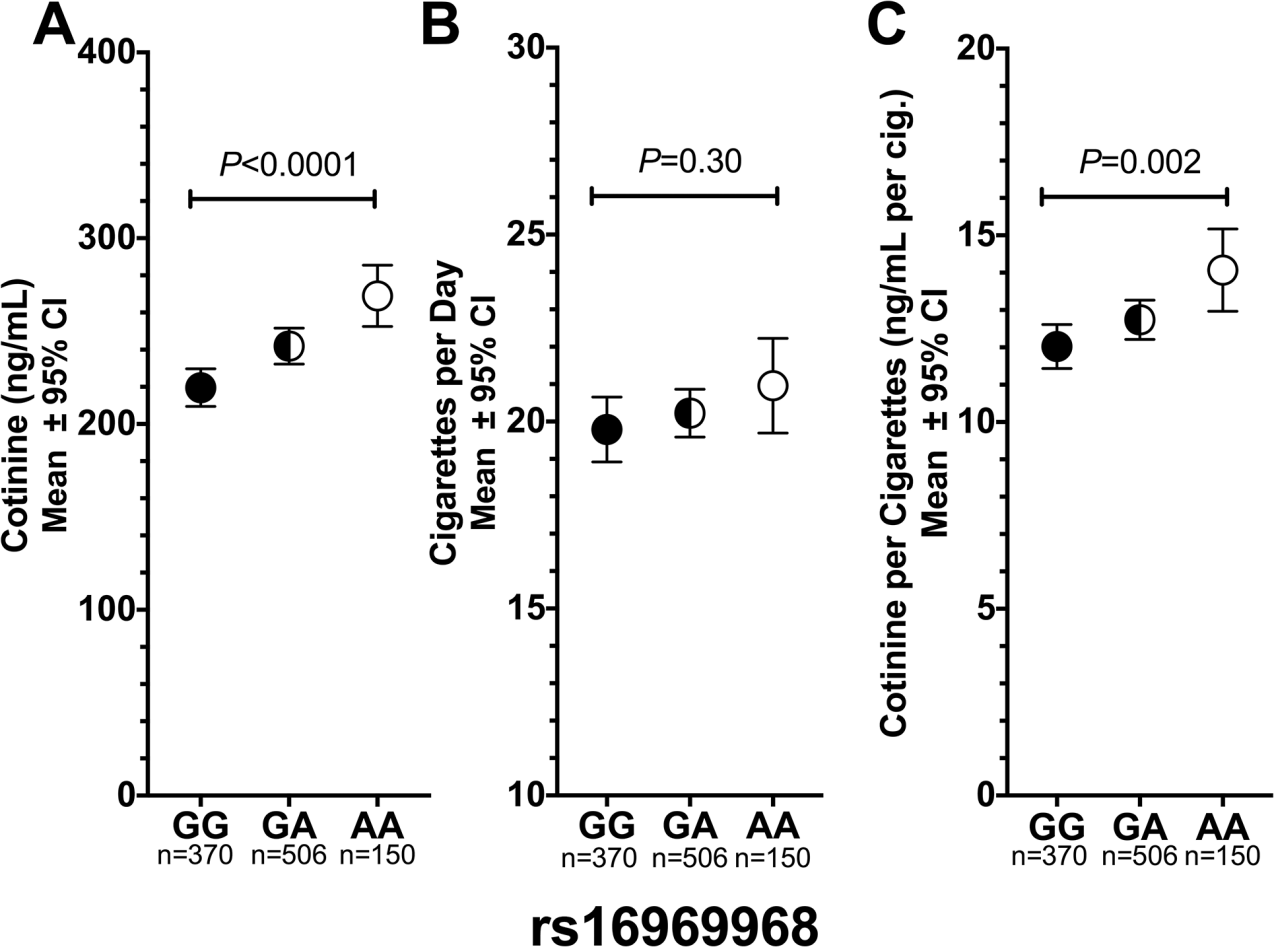
The association between *CHRNA5-A3-B4* variant rs16969968 and smoking behaviors among Caucasian smokers. (a) The ‘A’ allele of rs16969968 was significantly associated with higher cotinine levels (10.2% higher for GA and 22.5% higher for AA compared to GG). (b) No association between rs16969968 with cigarettes per day was observed. (c) The ‘A’ allele of rs16969968 was associated with significantly higher smoking intensity as indicated by cotinine per cigarette (6.0% higher for GA and 17.1% higher for AA compared to GG). Kruskal–Wallis tests were used for statistical comparisons. GG, GA, & AA represent different genotypes of rs16969968.

Among the screened participants, the ‘A’ allele of rs16969968 was significantly associated with 10% higher cotinine levels (per allele effect = 23.9 ng/mL, CI = 14.4–33.4, *P*<0.0001, Fig 1A), but not significantly with self-reported cigarettes per day (*P* = 0.30, Table 1 and Fig 1B). A significant association between rs16969968 and cotinine was also observed in the intent-to-treat participants (per allele effect = 22.4 ng/mL, CI = 6.7–38.1, *P* = 0.005). Interestingly, adjusting for self-reported cigarette consumption very modestly reduced the per allele effect of rs16969968 on cotinine (the per allele effect size of rs16969968 on cotinine was 21.8, CI = 12.6–30.9, P<0.0001 after adjusting for cigarettes per day versus the 23.9 ng/mL without adjustment, Fig 1C), indicating the weakness of self-reported consumption relative to cotinine as a measure of consumption. This suggests that smokers with the ‘A’ allele of rs16969968 likely extracted more nicotine per cigarette, which is supported by the observation that smokers with the ‘A’ allele of rs16969968 had significantly higher cotinine per cigarette (*P* = 0.002, Fig 1C). The association between rs16969968 and smoking behaviors did not differ significantly by gender (data not shown) as previously observed [4–7, 41].

#### rs588765

Among the screened participants, rs588765 was not significantly associated with cigarettes per day, cotinine levels, or cotinine per cigarette (Table 1 and S1 Fig). Adjusting for rs16969968 did not significantly alter the association between rs588765 and smoking phenotypes (data not shown).

#### rs578776

Among the screened participants, the ‘A’ allele of rs578776 was significantly associated with 8% lower cotinine levels (per allele effect is -18.9 ng/mL CI = -29.7 –-8.2, *P* = 0.002, Table 1 and S2 Fig), but not significantly associated with cigarettes per day (*P* = 0.53, Table 1 and S2 Fig). Adjusting for self-reported cigarette consumption did not substantially reduce the per allele effect size of rs578776 on cotinine levels (the per allele effect size of rs578776 was -18.4 ng/mL, CI = -28.8 –-8.1, *P*<0.0001 versus the -18.9 ng/mL without adjustment, S2 Fig). However, the association was no longer significant after adjusting for rs16969968 (the per allele effect size was -8.2 ng/mL, CI = -20.24–3.93, *P* = 0.19 versus the -18.9 ng/mL without adjustment).

#### Haplotype analyses

Among the screened participants, cigarettes per day and FTND levels did not significantly differ between the *CHRNA5-A3-B4* haplotypes (Table 2). However, haplotype 3 was significantly associated with lower cotinine levels compared to haplotype 1 (Table 2, P<0.0001).

**Table 2:** The association between *CHRNA5-A3-B4* rs16969968_rs588765 haplotype and baseline smoking behaviors in the screened population (N = 1026).

	β	95% CI	P
**CIGARETTES PER DAY**			
*CHRNA5-A3-B4* Haplotype			
Haplotype 1 (G_C)	Reference		
Haplotype 2 (G_T)	0.32	-0.60, 1.25	0.492
Haplotype 3 (A_C)	0.85	-0.07, 1.77	0.069
NMR (<0.31 vs. ≥0.31)	1.64	0.62, 2.65	**0.001**
**CO LEVELS**			
*CHRNA5-A3-B4* Haplotype			
Haplotype 1 (G_C)	Reference		
Haplotype 2 (G_T)	0.23	-1.39, 1.86	0.776
Haplotype 3 (A_C)	1.36	-0.30, 3.03	0.109
NMR (<0.31 vs. ≥0.31)	1.71	0.02, 3.40	0.048
**FTND**			
*CHRNA5-A3-B4* Haplotype			
Haplotype 1 (G_C)	Reference		
Haplotype 2 (G_T)	0.13	-0.11, 0.37	0.299
Haplotype 3 (A_C)	0.23	-0.079, 0.47	0.058
NMR (<0.31 vs. ≥0.31)	0.26	-0.072, 0.52	0.057
Cotinine *			
*CHRNA5-A3-B4* Haplotype			
Haplotype 1 (G_C)	Reference		
Haplotype 2 (G_T)	12.2	-0.39, 24.8	0.058
Haplotype 3 (A_C)	33.1	20.5, 45.7	**<0.001**

All models were adjusted for age and gender. NMR = nicotine metabolite ratio.

The *CHRNA5-A3-B4* haplotype was defined by rs16969968 and rs588765.

* Cotinine is a part of NMR, and cotinine levels are not directly comparable in people with different NMR [48], thus NMR was not included in the cotinine analysis.

### Association between *CHRNA5-A3-B4* SNPs and haplotype with smoking cessation

We next investigated the association between *CHRNA5-A3-B4* variants and smoking abstinence in the intent-to-treat group (N = 654). Overall at EOT, 19.8% of the participants (42 out of 212) receiving the placebo treatment were abstinent, 24.8% of the participants (55 out of 222) receiving the nicotine patch treatment were abstinent, and 38.6% of the participants (85 out of 220) receiving the varenicline treatment were abstinent.

#### rs16969968

**Fig 2:**
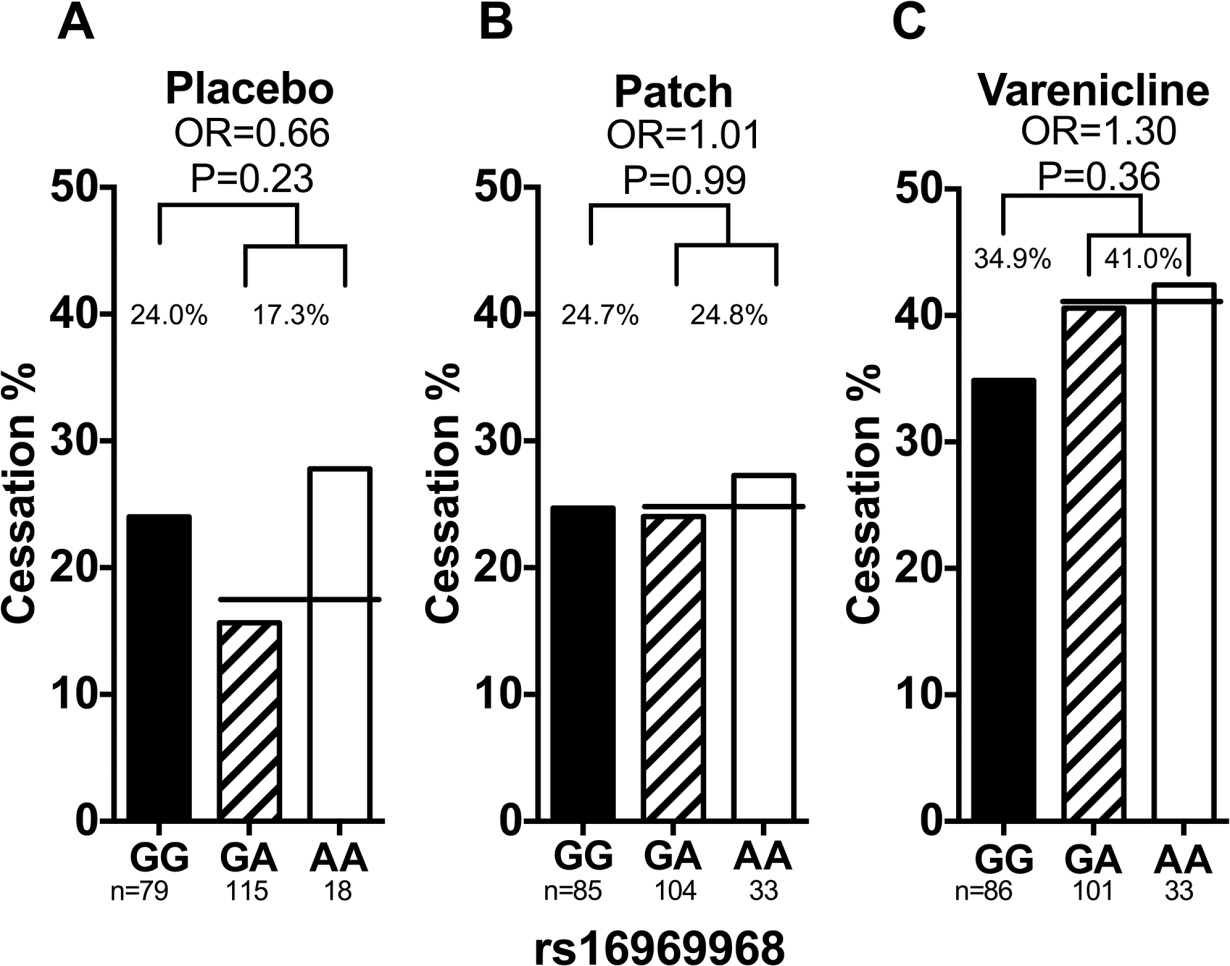
The association between rs16969968 and smoking abstinence in Caucasian smokers. (a) Rs16969968 was not associated with smoking abstinence in smokers treated with placebo. Adjusting for NMR, sex and age did not significantly alter the association (OR_adjusted_ = 0.66, P_adjusted_ = 0.24). (b) Rs16969968 was not associated with smoking abstinence in smokers treated with nicotine replacement patch. Adjusting for NMR, sex and age did not significantly alter the association (OR_adjusted_ = 0.99, P_adjusted_ = 0.98). (c) Rs16969968 was not associated with smoking abstinence in smokers treated with varenicline. Adjusting for NMR, sex and age did not significantly alter the association (OR_adjusted_ = 1.29, P_adjusted_ = 0.38). The black lines represent the average abstinence rate between the GA and AA group. Statistical significance was evaluated using dominant models (i.e. GG = 0 and GA/AA = 1). OR = odds ratio. NMR = nicotine metabolite ratio. GG, GA, & AA represent different genotypes of rs16969968.

Smoking abstinence rates by rs16969968 genotype are shown in Fig 2. rs16969968 was not significantly associated with smoking abstinence in the total (adjusting for treatment arm), placebo, nicotine patch, or varenicline groups (total odds ratio (OR) = 1.00, 95% CI = 0.70–1.42 and P = 0.99, placebo OR = 0.66, 95% CI = 0.33–1.31 and P = 0.23, patch OR = 1.01, 95% CI = 0.54–1.88 and P = 0.99, or varenicline OR = 1.30, 95% CI = 0.74–2.28 and P = 0.36). There were no significant two-way genotype-by-treatment interactions (S1 Table). These results were not altered substantially when adjusting for the rate of nicotine metabolism (dichotomized as NMR<0.31 and ≥ 0.31 [18]), age, sex, and baseline cigarette consumption (Fig 2).

#### rs588765

**Fig 3:**
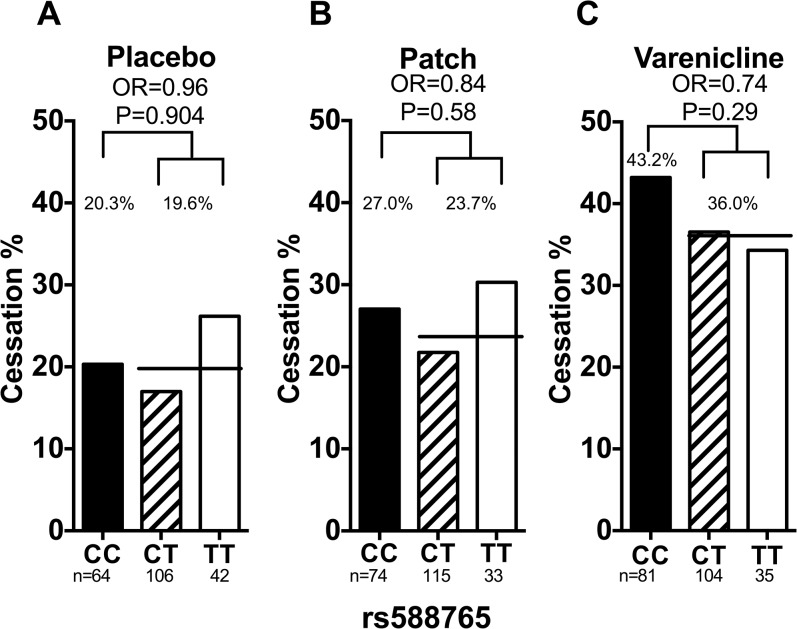
The association between rs588765 and smoking abstinence in Caucasian smokers. (a) Rs588765 was not associated with smoking abstinence in smokers treated with placebo. Adjusting for NMR, sex and age did not significantly alter the association (OR_adjusted_ = 0.97, P_adjusted_ = 0.93). (b) Rs588765 was not associated with smoking abstinence in smokers treated with nicotine replacement patch. Adjusting for NMR, sex and age did not significantly alter the association (OR_adjusted_ = 0.81, P_adjusted_ = 0.52). (c) Rs588765 was not associated with smoking abstinence in smokers treated with varenicline. Adjusting for NMR, sex and age did not significantly alter the association (OR_adjusted_ = 0.77, P_adjusted_ = 0.36). The black lines represent the average abstinence rate between the CT and TT group. Statistical significance was evaluated using dominant models (i.e. CC = 0 and CT/TT = 1). OR = odds ratio. CC, CT, & TT represent different genotypes of rs588765.

Smoking abstinence rates by rs588765 genotype are shown in Fig 3. rs588765 was not significantly associated with smoking abstinence in the total (adjusting for treatment arm), placebo, nicotine patch group, or varenicline groups (total OR = 0.82, 95% CI = 0.57–1.18 and P = 0.29, placebo OR = 0.96, 95% CI = 0.46–1.99 and P = 0.90, patch OR = 0.84, 95% CI = 0.44–1.58 and P = 0.58, and varenicline OR = 0.74 95% CI = 0.42–1.29 P = 0.29). There were no significant two-way genotype-by-treatment interactions (S2 Table). These results were not altered substantially when adjusted for the rate of nicotine metabolism, age, sex, and cigarette consumption (Fig 3).

#### rs578776

**Fig 4:**
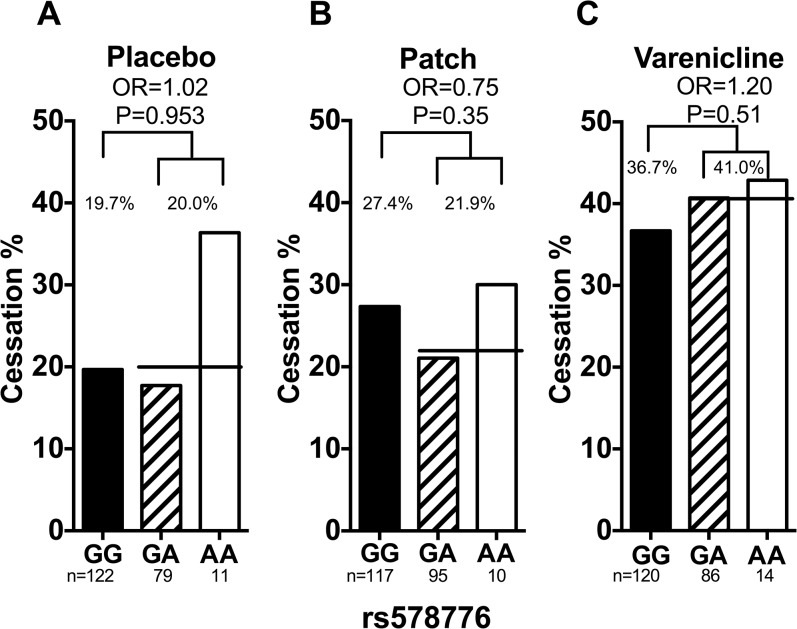
The association between rs578776 and smoking abstinence in Caucasian smokers. (a) Rs578776 was not associated with smoking abstinence in smokers treated with placebo. Adjusting for NMR, sex and age did not significantly alter the association (OR_adjusted_ = 1.00, P_adjusted_ = 0.99). (b) Rs578776 was not associated with smoking abstinence in smokers treated with nicotine replacement patch. Adjusting for NMR, sex and age did not significantly alter the association (OR_adjusted_ = 0.74, P_adjusted_ = 0.35). (c) Rs578776 was not associated with smoking abstinence in smokers treated with varenicline. Adjusting for NMR, sex and age did not significantly alter the association (OR_adjusted_ = 1.14, P_adjusted_ = 0.63). The black lines represent the average abstinence rate between the GA and AA group. Statistical significance was evaluated using dominant models (i.e. GG = 0 and GA/AA = 1). OR = odds ratio. NMR = nicotine metabolite ratio. GG, GA, & AA represent different genotypes of rs578776.

Smoking abstinence rates by rs578776 genotype are shown in Fig 4. rs578776 was not significantly associated with smoking abstinence in the total (adjusting for treatment arm), placebo, nicotine patch, or varenicline groups (total OR = 0.98, 95% CI = 0.69–1.40 and P = 0.93, placebo OR = 1.02, 95% CI = 0.52–2.02 and P = 0.95, patch OR = 0.75, 95% CI = 0.40–1.38 and P = 0.35, and varenicline OR = 1.20, 95% CI = 0.70–2.07 and P = 0.51). There were no significant genotype-by-treatment interactions (S3 Table). These results were not altered substantially when adjusted for the rate of nicotine metabolism, age, sex, and cigarette consumption (Fig 4).

#### Haplotype analyses

**Fig 5:**
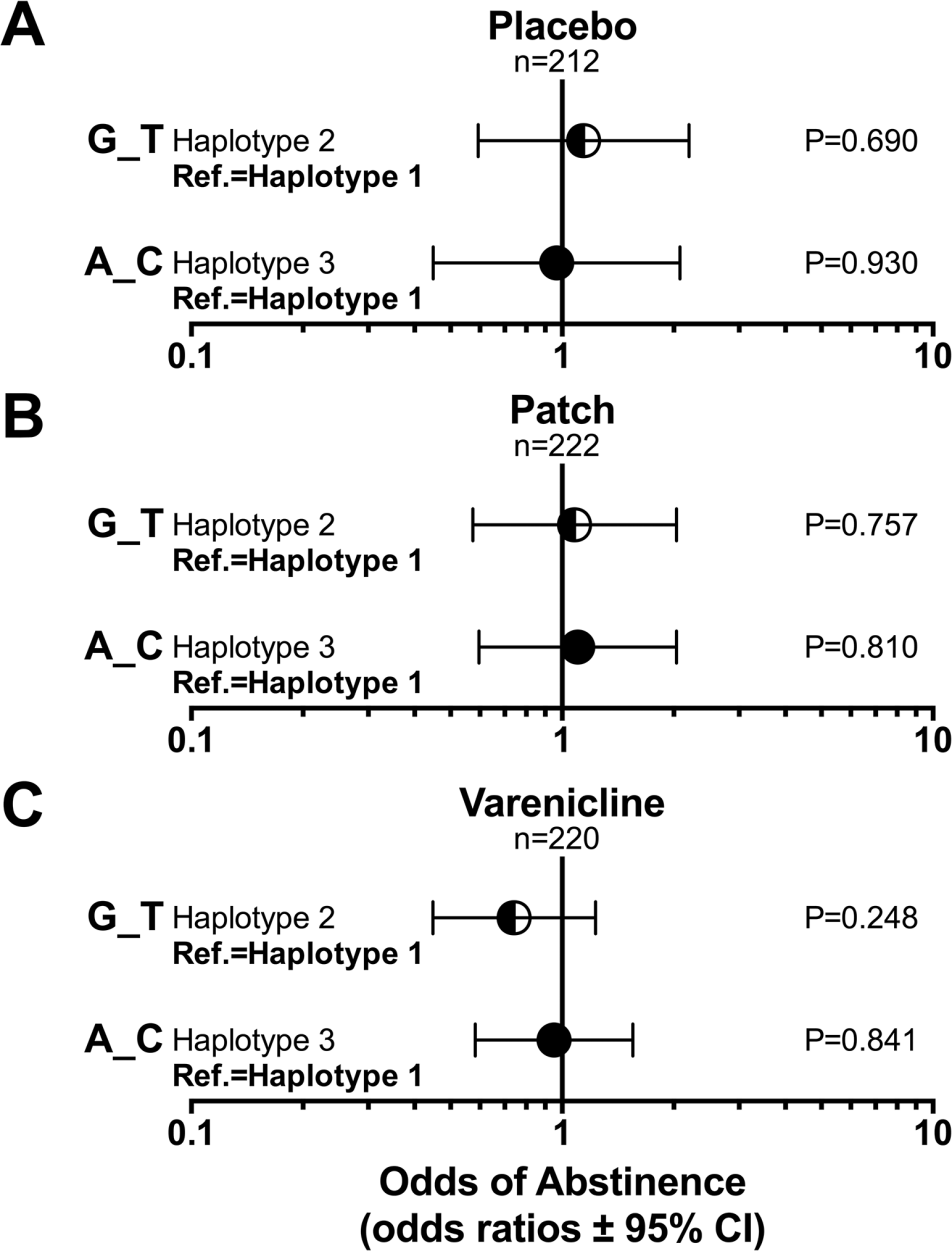
The association between *CHRNA5-A3-B4* haplotype and smoking abstinence. (a) *CHRNA5-A3-B4* haplotype was not associated with smoking abstinence in smokers treated with placebo. Adjusting for NMR, sex and age did not significantly alter the association (Haplotype 2: OR_adjusted_ = 1.21, P_adjusted_ = 0.58; Haplotype 3: OR_adjusted_ = 1.02, P_adjusted_ = 0.96). (b) *CHRNA5-A3-B4* haplotype was not associated with smoking abstinence in smokers treated with nicotine replacement patch. Adjusting for NMR, sex and age did not significantly alter the association (Haplotype 2: OR_adjusted_ = 1.06, P_adjusted_ = 0.85; Haplotype 3: OR_adjusted_ = 1.09, P_adjusted_ = 0.78). (c) *CHRNA5-A3-B4* haplotype was not associated with smoking abstinence in smokers treated with varenicline. Adjusting for NMR, sex and age did not significantly alter the association (Haplotype 2: OR_adjusted_ = 0.78, P_adjusted_ = 0.34; Haplotype 3: OR_adjusted_ = 0.98, P_adjusted_ = 0.93). The *CHRNA5-A3-B4* haplotype was defined by rs16969968 and rs588765. OR = odds ratio. NMR = nicotine metabolite ratio.

Smoking abstinence rates by rs16969968_rs588765 haplotype are shown in Fig 5. In logistic regression models, haplotype 2 (G_T) and haplotype 3 (G_C) were not significantly associated with smoking abstinence within each treatment group independently compared to the reference haplotype 1 group (G_C) (OR = 0.74–1.16, P>0.05). Furthermore, the haplotypes did not predict abstinence in the entire study group, and no significant haplotype-by-treatment interactions were observed (Table 3 and S3 Fig.).

**Table 3:** The association between *CHRNA5-A3-B4* Haplotype and end of treatment smoking abstinence in the intent to treat population (N = 654).

	Effect on Abstinence at End of Treatment		
PREDICTORS	Odds Ratio	95% CI	P
*Haplotype*			
Haplotype 1 (G_C)	Reference		
Haplotype 2 (G_T)	0. 98	0.46, 2.1	0.964
Haplotype 3 (A_C)	1.15	0.60, 2.2	0.679
*Treatment*			
Placebo	Reference		
Active Treatment	2.4	0.70, 8.23	0.165
*Interaction of haplotype and treatment*			
Haplotype 1 and active treatment	Reference		
Haplotype 2 and active treatment	1.01	0.43, 2.36	0.985
Haplotype 3 and active treatment	0.75	0.35, 1.60	0.457

All models were adjusted for age, gender and nicotine metabolism.

The *CHRNA5-A3-B4* haplotype was defined by rs16969968 and rs588765 as previously defined [30].

#### 6 month and 12 month smoking abstinence

*CHRNA5-A3-B4* variants rs16969968, rs588765, & rs578776 were not significantly associated with smoking abstinence at 6 month and 12 month follow-up (ORs range from 0.54 to 1.98, P values range from 0.11 to 0.92, S4 Table). Similarly, rs16969968_rs588765 haplotypes were not significantly associated with smoking abstinence at 6 month and 12 month follow-up (S5 and S6 Tables).

## Discussion

In this study, we report a lack of association between *CHRNA5-A3-B4* genetic variants/haplotype and end of treatment smoking cessation outcomes in Caucasian smokers despite the expected significant association between *CHRNA5-A3-B4* genetic variants and baseline smoking behaviors (cotinine levels). Our study is the first placebo-controlled clinical trial investigating the association of *CHRNA5-A3-B4* genetic variants with smoking cessation during treatment with the nicotine patch versus varenicline. We observed no significant *CHRNA5-A3-B4* genotype-by-treatment interactions with or without adjusting for the rate of nicotine metabolism (i.e. CYP2A6 activity). Together, these findings would not support the use of *CHRNA5-A3-B4* genetic variants to optimize smoking cessation pharmacotherapy selection in Caucasian smokers. Our findings are in agreement with previous large genome-wide association studies which reported that *CHRNA5-A3-B4* variants on chromosome 15 are associated with tobacco consumption among smokers but not smoking status (i.e., current smoker vs. former smoker, n = 41,278), indicating *CHRNA5-A3-B4* variants are not associated with smoking cessation [5]. Our findings also agree with the conclusions of the Munafò and colleagues study, which suggested the clinical significance of *CHRNA5-A3-B4* genetic variants on smoking cessation was limited [21].

On the other hand, our findings are in contrast to those findings reported by Chen and colleagues and Bergen and colleagues, who reported significant associations between *CHRNA5-A3-B4* haplotype and smoking cessation in smokers who received placebo treatment [30, 31]. It is worth noting that both of these previous studies were partial secondary analyses of an overlapping clinical trial sample, which had significant heterogeneity in the active treatment group (they had 5 distinct active treatments) and Chen et al., had a relatively small placebo group (n = 132) in contrast to n = 212 in the current study [18]. However, since the *CHRNA5-A3-B4* associations with smoking cessation were primarily observed in their placebo group, it is unclear why the findings differed between our study and these past studies. The current lack of association between *CHRNA5-A3-B4* and smoking cessation was not influenced by the rate of nicotine metabolism (i.e. adjusting for NMR) and a follow-up study by Chen and colleagues reported that *CHRNA5-A3-B4* and *CYP2A6* genotype estimated rate of nicotine metabolism were independent signals [37]. Together this suggests that it is unlikely that the lack of association between *CHRNA5-A3-B4* and smoking cessation observed in the current study is related to differences in nicotine metabolism.

Smoking cessation is an important and complex smoking phenotype; genetic factors are one of many factors which can influence smoking cessation. One possibility for the discrepancy is the variability in participant demographics across studies. The clinical trial reported in past studies consisted of 58% females, whereas our study had 42% females. All studies followed available guidelines for pharmacotherapy administration. However, it is possible that the content or intensity of behavioral counseling differed across studies. If such contextual factors could alter genetic association results, then it could be argued that the genetic findings are unlikely to be sufficiently robust for use in clinical practice.

To demonstrate potential clinical utility in treatment decision-making, a significant gene-by-treatment interaction is important; however, we did not observe any evidence or trends in this regard. Recently we have reported that varenicline is more efficacious than nicotine patch in smokers with a faster rate of nicotine metabolism in contrast to smokers with a slower rate of nicotine metabolism [18]. Hence, the rate of nicotine metabolism can be used to predict the most efficacious type of smoking cessation therapy. In the case of *CHRNA5-A3-B4* variants, the existing literature is conflicting, lacking a consistent genotype-by-treatment interaction [19–21, 30, 31]. Therefore, it is unlikely that *CHRNA5-A3-B4* can be used clinically to predict the most effective smoking cessation pharmacotherapy.

Our conclusions should be interpreted in the context of statistical power. However, in the model of cessation in the full sample of 654 individuals adjusting for treatment arm, the ORs for the *CHRNA5-A3-B4* variants differed very little from 1, suggesting no trends for association. For a treatment arm specific effect, at the current samples size (n = 212 to 222 per arm), we are at 0.8 power to detect any effect size larger than OR = ~2.3 for any genetic variants with a 40% allele frequency. Therefore, our data can statistically support that *CHRNA5-A3-B4* variants do not have a large impact on smoking cessation. Although it is possible that our study was not sufficiently powered to reject a weak association between *CHRNA5-A3-B4* variants and smoking cessation, the absence of clinically meaningful trends in our study and the lack of genotype-by-treatment interactions reported by previous studies would still support our conclusion.

In contrast to the inconsistent reports on associations with cessation, gene variants in *CHRNA5-A3-B4*, particularly rs16969968 and correlated SNPs, have been consistently associated with tobacco consumption in Caucasian smokers [4–7, 11, 12]. In this study, we observed a significant association between rs16969968 and cotinine levels. The ‘A’ allele of rs16969968 was associated with a roughly 23.9 ng/ml increase in cotinine per allele. These effect sizes were within the previously reported range of 24–100 ng/ml per allele [42–44]. In contrast, we did not observe any significant association between *CHRNA5-A3-B4* variants and cigarettes per day despite the significant association between nicotine metabolism rate and cigarettes per day. A number of previous studies have also found that the rate of nicotine metabolism has a greater influence on cigarette consumption (i.e. cigarettes smoked per day, total nicotine equivalents) compared to *CHRNA5-A3-B4* gene variants [44, 45]. Thus, it is interesting that *CHRNA5-A3-B4* variants generally have a greater effect on lung cancer risk than variation in nicotine metabolism [45]. This observation suggests that the *CHRNA5-A3-B4* gene variants may modulate lung cancer risk by mechanisms in addition to their effects on smoking behavior, such as directly altering cell proliferation and survival [46]. This hypothesis could be directly tested by measuring better tobacco consumption biomarkers (such as urinary total nicotine equivalents) in lung cancer epidemiology studies [42, 47].

In conclusion, the findings of our study, in conjunction with some previous reports, demonstrated a lack of *CHRNA5-A3-B4* genotype by active smoking cessation pharmacotherapy interaction in smokers, and no effect within any treatment arm including placebo. Thus these current and previous data together suggest that it is unlikely that *CHRNA5-A3-B4* could be used clinically to predict the most effective smoking cessation pharmacotherapy to offer to the individual smoker seeking to quit.

## Supporting Information

S1 Fig The association between *CHRNA5-A3-B4* variant rs588765 and smoking behaviors among Caucasian smokers. (a) No association between rs588765 with cotinine levels was observed. (b) No association between rs588765 with cigarettes per day was observed. (c) No association between rs588765 with smoking intensity (as indicated by cotinine per cigarette) was observed. Kruskal–Wallis tests were used for statistical comparisons.
(DOCX)

S2 Fig The association between *CHRNA5-A3-B4* variant rs578776 and smoking behaviors among Caucasian smokers. (a) The ‘A’ allele of rs578776 was associated with significantly lower cotinine levels. (b) No association between rs578776 with cigarettes per day was observed. (c) The ‘A’ allele of rs578776 was associated with significantly lower smoking intensity as indicated by cotinine per cigarette. Kruskal–Wallis tests were used for statistical comparisons.
(DOCX)

S3 Fig The association between *CHRNA5-A3-B4* haplotype rs16969968_rs588765 and smoking abstinence at end of treatment. (DOCX)

S1 Table The two-way interaction table for rs16969968 on smoking cessation. (DOCX)

S2 Table The two-way interaction table for rs588765 on smoking cessation. (DOCX)

S3 Table The two way interaction table for rs578776 on smoking cessation. (DOCX)

S4 Table The association between *CHRNA5-A3-B4* variants and 6 month and 12 month smoking abstinence in the intent to treat population (N = 654) (DOCX)

S5 Table The association between *CHRNA5-A3-B4* Haplotype and 6 month smoking abstinence in the intent to treat population (N = 654). (DOCX)

S6 Table The association between *CHRNA5-A3-B4* Haplotype and 12 month smoking abstinence in the intent to treat population (N = 654). (DOCX)

## References

[1] World Health Organization. Who report on the global tobacco epidemic, 2011: Warning about the dangers of tobacco. 2011.

[2] Jha P, Ramasundarahettige C, Landsman V, Rostron B, Thun M, Anderson RN, et al 21st-century hazards of smoking and benefits of cessation in the United States. N Engl J Med. 2013;368(4):341–50. Epub 2013/01/25. 10.1056/NEJMsa1211128 . 23343063

[3] Benowitz NL. Nicotine addiction. N Engl J Med. 2010;362(24):2295–303. Epub 2010/06/18. 10.1056/NEJMra0809890 20554984 PMC2928221

[4] Thorgeirsson TE, Geller F, Sulem P, Rafnar T, Wiste A, Magnusson KP, et al A variant associated with nicotine dependence, lung cancer and peripheral arterial disease. Nature. 2008;452(7187):638–42. Epub 2008/04/04. 10.1038/nature06846 . 18385739 PMC4539558

[5] Consortium TTaG. Genome-wide meta-analyses identify multiple loci associated with smoking behavior. Nature genetics. 2010;42(5):441–7. Epub 2010/04/27. 10.1038/ng.571 20418890 PMC2914600

[6] Thorgeirsson TE, Gudbjartsson DF, Surakka I, Vink JM, Amin N, Geller F, et al Sequence variants at CHRNB3-CHRNA6 and CYP2A6 affect smoking behavior. Nat Genet. 2010;42(5):448–53. Epub 2010/04/27. doi: ng.573 [pii] 10.1038/ng.573 20418888 PMC3080600

[7] Liu JZ, Tozzi F, Waterworth DM, Pillai SG, Muglia P, Middleton L, et al Meta-analysis and imputation refines the association of 15q25 with smoking quantity. Nat Genet. 2010;42(5):436–40. Epub 2010/04/27. 10.1038/ng.572 . 20418889 PMC3612983

[8] Pillai SG, Ge D, Zhu G, Kong X, Shianna KV, Need AC, et al A genome-wide association study in chronic obstructive pulmonary disease (COPD): identification of two major susceptibility loci. PLoS Genet. 2009;5(3):e1000421 Epub 2009/03/21. 10.1371/journal.pgen.1000421 19300482 PMC2650282

[9] Siedlinski M, Cho MH, Bakke P, Gulsvik A, Lomas DA, Anderson W, et al Genome-wide association study of smoking behaviours in patients with COPD. Thorax. 2011;66(10):894–902. Epub 2011/06/21. 10.1136/thoraxjnl-2011-200154 21685187 PMC3302576

[10] Saccone SF, Hinrichs AL, Saccone NL, Chase GA, Konvicka K, Madden PA, et al Cholinergic nicotinic receptor genes implicated in a nicotine dependence association study targeting 348 candidate genes with 3713 SNPs. Hum Mol Genet. 2007;16(1):36–49. Epub 2006/12/01. 10.1093/hmg/ddl438 17135278 PMC2270437

[11] Saccone NL, Schwantes-An TH, Wang JC, Grucza RA, Breslau N, Hatsukami D, et al Multiple cholinergic nicotinic receptor genes affect nicotine dependence risk in African and European Americans. Genes, brain, and behavior. 2010;9(7):741–50. Epub 2010/06/30. 10.1111/j.1601-183X.2010.00608.x 20584212 PMC2970751

[12] Saccone NL, Culverhouse RC, Schwantes-An TH, Cannon DS, Chen X, Cichon S, et al Multiple independent loci at chromosome 15q25.1 affect smoking quantity: a meta-analysis and comparison with lung cancer and COPD. PLoS Genet. 2010;6(8). Epub 2010/08/12. 10.1371/journal.pgen.1001053 20700436 PMC2916847

[13] Gonzales D, Rennard SI, Nides M, Oncken C, Azoulay S, Billing CB, et al Varenicline, an alpha4beta2 nicotinic acetylcholine receptor partial agonist, vs sustained-release bupropion and placebo for smoking cessation: a randomized controlled trial. JAMA. 2006;296(1):47–55. Epub 2006/07/06. doi: 296/1/47 [pii]. 10.1001/jama.296.1.47 . 16820546

[14] Xian H, Scherrer JF, Madden PA, Lyons MJ, Tsuang M, True WR, et al The heritability of failed smoking cessation and nicotine withdrawal in twins who smoked and attempted to quit. Nicotine Tob Res. 2003;5(2):245–54. Epub 2003/05/15. . 12745498

[15] Cahill K, Stevens S, Perera R, Lancaster T. Pharmacological interventions for smoking cessation: an overview and network meta-analysis. Cochrane Database Syst Rev. 2013;5:CD009329. Epub 2013/06/04. 10.1002/14651858.CD009329.pub2 . 23728690 PMC8406789

[16] Stead LF, Perera R, Bullen C, Mant D, Hartmann-Boyce J, Cahill K, et al Nicotine replacement therapy for smoking cessation. Cochrane Database Syst Rev. 2012;11:CD000146. 10.1002/14651858.CD000146.pub4 . 23152200

[17] Lessov CN, Martin NG, Statham DJ, Todorov AA, Slutske WS, Bucholz KK, et al Defining nicotine dependence for genetic research: evidence from Australian twins. Psychological medicine. 2004;34(5):865–79. . 15500307 10.1017/s0033291703001582

[18] Lerman C, Schnoll RA, Hawk LW Jr., Cinciripini P, George TP, Wileyto EP, et al Use of the nicotine metabolite ratio as a genetically informed biomarker of response to nicotine patch or varenicline for smoking cessation: a randomised, double-blind placebo-controlled trial. The lancet Respiratory medicine. 2015;3(2):131–8. 10.1016/S2213-2600(14)70294-2 . 25588294 PMC4480925

[19] Baker TB, Weiss RB, Bolt D, von Niederhausern A, Fiore MC, Dunn DM, et al Human neuronal acetylcholine receptor A5-A3-B4 haplotypes are associated with multiple nicotine dependence phenotypes. Nicotine Tob Res. 2009;11(7):785–96. 10.1093/ntr/ntp064 19436041 PMC2699926

[20] Freathy RM, Ring SM, Shields B, Galobardes B, Knight B, Weedon MN, et al A common genetic variant in the 15q24 nicotinic acetylcholine receptor gene cluster (CHRNA5-CHRNA3-CHRNB4) is associated with a reduced ability of women to quit smoking in pregnancy. Hum Mol Genet. 2009;18(15):2922–7. 10.1093/hmg/ddp216 19429911 PMC2706684

[21] Munafo MR, Johnstone EC, Walther D, Uhl GR, Murphy MF, Aveyard P. CHRNA3 rs1051730 genotype and short-term smoking cessation. Nicotine Tob Res. 2011;13(10):982–8. Epub 2011/06/22. 10.1093/ntr/ntr106 21690317 PMC3179672

[22] Sarginson JE, Killen JD, Lazzeroni LC, Fortmann SP, Ryan HS, Schatzberg AF, et al Markers in the 15q24 nicotinic receptor subunit gene cluster (CHRNA5-A3-B4) predict severity of nicotine addiction and response to smoking cessation therapy. Am J Med Genet B Neuropsychiatr Genet. 2011;156B(3):275–84. Epub 2011/01/27. 10.1002/ajmg.b.31155 . 21268243

[23] Zhu AZ, Zhou Q, Cox LS, David SP, Ahluwalia JS, Benowitz NL, et al Association of CHRNA5-A3-B4 SNP rs2036527 With Smoking Cessation Therapy Response in African-American Smokers. Clin Pharmacol Ther. 2014;96(2):256–65. Epub 2014/04/16. 10.1038/clpt.2014.88 24733007 PMC4111775

[24] Breitling LP, Twardella D, Hoffmann MM, Witt SH, Treutlein J, Brenner H. Prospective association of dopamine-related polymorphisms with smoking cessation in general care. Pharmacogenomics. 2010;11(4):527–36. Epub 2010/03/31. 10.2217/pgs.10.1 . 20350135

[25] Conti DV, Lee W, Li D, Liu J, Van Den Berg D, Thomas PD, et al Nicotinic acetylcholine receptor beta2 subunit gene implicated in a systems-based candidate gene study of smoking cessation. Hum Mol Genet. 2008;17(18):2834–48. 10.1093/hmg/ddn181 18593715 PMC2525499

[26] Swan GE, Javitz HS, Jack LM, Wessel J, Michel M, Hinds DA, et al Varenicline for smoking cessation: nausea severity and variation in nicotinic receptor genes. Pharmacogenomics J. 2012;12(4):349–58. 10.1038/tpj.2011.19 21606948 PMC3405554

[27] Uhl GR, Drgon T, Johnson C, Ramoni MF, Behm FM, Rose JE. Genome-wide association for smoking cessation success in a trial of precessation nicotine replacement. Mol Med. 2010;16(11–12):513–26. Epub 2010/09/03. 10.2119/molmed.2010.00052 20811658 PMC2972392

[28] Uhl GR, Liu QR, Drgon T, Johnson C, Walther D, Rose JE. Molecular genetics of nicotine dependence and abstinence: whole genome association using 520,000 SNPs. BMC genetics. 2007;8:10 Epub 2007/04/05. 10.1186/1471-2156-8-10 17407593 PMC1853105

[29] Uhl GR, Liu QR, Drgon T, Johnson C, Walther D, Rose JE, et al Molecular genetics of successful smoking cessation: convergent genome-wide association study results. Arch Gen Psychiatry. 2008;65(6):683–93. Epub 2008/06/04. 10.1001/archpsyc.65.6.683 18519826 PMC2430596

[30] Chen LS, Baker TB, Piper ME, Breslau N, Cannon DS, Doheny KF, et al Interplay of genetic risk factors (CHRNA5-CHRNA3-CHRNB4) and cessation treatments in smoking cessation success. Am J Psychiatry. 2012;169(7):735–42. Epub 2012/06/01. 10.1176/appi.ajp.2012.11101545 22648373 PMC3433845

[31] Bergen AW, Javitz HS, Krasnow R, Nishita D, Michel M, Conti DV, et al Nicotinic acetylcholine receptor variation and response to smoking cessation therapies. Pharmacogenet Genomics. 2013;23(2):94–103. Epub 2012/12/20. 10.1097/FPC.0b013e32835cdabd 23249876 PMC3563676

[32] SRNT Subcommittee on Biochemical Verification. Biochemical verification of tobacco use and cessation. Nicotine Tob Res. 2002;4(2):149–59. Epub 2002/05/25. 10.1080/14622200210123581 . 12028847

[33] Purcell S, Neale B, Todd-Brown K, Thomas L, Ferreira MA, Bender D, et al PLINK: a tool set for whole-genome association and population-based linkage analyses. Am J Hum Genet. 2007;81(3):559–75. 10.1086/519795 17701901 PMC1950838

[34] Lea RA, Dickson S, Benowitz NL. Within-subject variation of the salivary 3HC/COT ratio in regular daily smokers: prospects for estimating CYP2A6 enzyme activity in large-scale surveys of nicotine metabolic rate. J Anal Toxicol. 2006;30(6):386–9. Epub 2006/07/29. . 16872570 10.1093/jat/30.6.386

[35] St Helen G, Novalen M, Heitjan DF, Dempsey D, Jacob P 3rd, Aziziyeh A, et al Reproducibility of the nicotine metabolite ratio in cigarette smokers. Cancer Epidemiol Biomarkers Prev. 2012;21(7):1105–14. Epub 2012/05/04. 10.1158/1055-9965.EPI-12-0236 22552800 PMC3392523

[36] Zhu AZ, Zhou Q, Cox LS, Ahluwalia JS, Benowitz NL, Tyndale RF. Variation in trans-3'-hydroxycotinine glucuronidation does not alter the nicotine metabolite ratio or nicotine intake. PLoS One. 2013;8(8):e70938 Epub 2013/08/13. 10.1371/journal.pone.0070938 23936477 PMC3732272

[37] Chen LS, Bloom AJ, Baker TB, Smith SS, Piper ME, Martinez M, et al. Pharmacotherapy Effects on Smoking Cessation Vary with Nicotine Metabolism Gene (CYP2A6). Addiction. 2013. Epub 2013/09/17. 10.1111/add.12353 . 24033696 PMC3946972

[38] Jacob P 3rd, Yu L, Duan M, Ramos L, Yturralde O, Benowitz NL. Determination of the nicotine metabolites cotinine and trans-3'-hydroxycotinine in biologic fluids of smokers and non-smokers using liquid chromatography-tandem mass spectrometry: biomarkers for tobacco smoke exposure and for phenotyping cytochrome P450 2A6 activity. J Chromatogr B Analyt Technol Biomed Life Sci. 2011;879(3–4):267–76. 10.1016/j.jchromb.2010.12.012 21208832 PMC3050598

[39] Tanner J, Novalen M, Jatlow P, Huestis M, Murphy S, Kaprio J, et al. Nicotine Metabolite Ratio (3-hydroxycotinine/cotinine) in Plasma and Urine by Different Analytical Methods and Laboratories: Implications for Clinical Implementation. Cancer Epidemiol Biomarkers Prev. 2015;In press. 10.1158/1055-9965.EPI-14-1381 PMC4526326 26014804

[40] Saccone NL, Wang JC, Breslau N, Johnson EO, Hatsukami D, Saccone SF, et al The CHRNA5-CHRNA3-CHRNB4 nicotinic receptor subunit gene cluster affects risk for nicotine dependence in African-Americans and in European-Americans. Cancer research. 2009;69(17):6848–56. Epub 2009/08/27. 10.1158/0008-5472.CAN-09-0786 19706762 PMC2874321

[41] Amos CI, Wu X, Broderick P, Gorlov IP, Gu J, Eisen T, et al Genome-wide association scan of tag SNPs identifies a susceptibility locus for lung cancer at 15q25.1. Nat Genet. 2008;40(5):616–22. Epub 2008/04/04. 10.1038/ng.109 18385676 PMC2713680

[42] Munafo MR, Timofeeva MN, Morris RW, Prieto-Merino D, Sattar N, Brennan P, et al Association between genetic variants on chromosome 15q25 locus and objective measures of tobacco exposure. J Natl Cancer Inst. 2012;104(10):740–8. Epub 2012/04/27. 10.1093/jnci/djs191 22534784 PMC3352832

[43] Keskitalo K, Broms U, Heliovaara M, Ripatti S, Surakka I, Perola M, et al Association of serum cotinine level with a cluster of three nicotinic acetylcholine receptor genes (CHRNA3/CHRNA5/CHRNB4) on chromosome 15. Human molecular genetics. 2009;18(20):4007–12. Epub 2009/07/25. 10.1093/hmg/ddp322 19628476 PMC2748889

[44] Zhu AZ, Renner CC, Hatsukami DK, Benowitz NL, Tyndale RF. CHRNA5-A3-B4 genetic variants alter nicotine intake and interact with tobacco use to influence body weight in Alaska Native tobacco users. Addiction. 2013;108(10):1818–28. Epub 2013/05/23. 10.1111/add.12250 23692359 PMC3775934

[45] Wassenaar CA, Dong Q, Wei Q, Amos CI, Spitz MR, Tyndale RF. Relationship between CYP2A6 and CHRNA5-CHRNA3-CHRNB4 variation and smoking behaviors and lung cancer risk. J Natl Cancer Inst. 2011;103(17):1342–6. Epub 2011/07/13. 10.1093/jnci/djr237 21747048 PMC3168937

[46] Brennan P, Hainaut P, Boffetta P. Genetics of lung-cancer susceptibility. Lancet Oncol. 2011;12(4):399–408. Epub 2010/10/19. 10.1016/S1470-2045(10)70126-1 . 20951091

[47] Spitz MR, Amos CI, Bierut LJ, Caporaso NE. Cotinine conundrum—a step forward but questions remain. J Natl Cancer Inst. 2012;104(10):720–2. Epub 2012/04/27. 10.1093/jnci/djs211 22534783 PMC3352835

[48] Zhu AZ, Renner CC, Hatsukami DK, Swan GE, Lerman C, Benowitz NL, et al The ability of plasma cotinine to predict nicotine and carcinogen exposure is altered by differences in CYP2A6: the influence of genetics, race, and sex. Cancer Epidemiol Biomarkers Prev. 2013;22(4):708–18. Epub 2013/02/02. 10.1158/1055-9965.EPI-12-1234-T 23371292 PMC3617060

