# Supplementary material for: Lack of Associations of CHRNA5-A3-B4 Genetic Variants with Smoking Cessation Treatment Outcomes in Caucasian Smokers despite Associations with Baseline Smoking
Source: PLoS One. 2015 May 26;10(5):e0128109. doi: 10.1371/journal.pone.0128109 (PMC4444267; doi:10.1371/journal.pone.0128109)
Supplement: S1 Fig — (a) No association between rs588765 with cotinine levels was observed. (b) No association between rs588765 with cigarettes per day was observed. (c) No association between rs588765 with smoking intensity (as indicated by cotinine per cigarette) was observed. Kruskal–Wallis tests were used for statistical comparisons. (DOCX) [file pone.0128109.s001.docx]

**S1 Fig.** The association between *CHRNA5-A3-B4* variant rs588765 and smoking behaviors among Caucasian smokers. (a) No association between rs588765 with cotinine levels was observed. (b) No association between rs588765 with cigarettes per day was observed. (c) No association between rs588765 with smoking intensity (as indicated by cotinine per cigarette) was observed. Kruskal–Wallis tests were used for statistical comparisons.
