# Supplementary material for: Lack of Associations of CHRNA5-A3-B4 Genetic Variants with Smoking Cessation Treatment Outcomes in Caucasian Smokers despite Associations with Baseline Smoking
Source: PLoS One. 2015 May 26;10(5):e0128109. doi: 10.1371/journal.pone.0128109 (PMC4444267; doi:10.1371/journal.pone.0128109)
Supplement: S2 Fig — (a) The ‘A’ allele of rs578776 was associated with significantly lower cotinine levels. (b) No association between rs578776 with cigarettes per day was observed. (c) The ‘A’ allele of rs578776 was associated with significantly lower smoking intensity as indicated by cotinine per cigarette. Kruskal–Wallis tests were used for statistical comparisons. (DOCX) [file pone.0128109.s002.docx]

**S2 Fig**. The association between *CHRNA5-A3-B4* variant rs578776 and smoking behaviors among Caucasian smokers. (a) The ‘A’ allele of rs578776 was associated with significantly lower cotinine levels. (b) No association between rs578776 with cigarettes per day was observed. (c) The ‘A’ allele of rs578776 was associated with significantly lower smoking intensity as indicated by cotinine per cigarette. Kruskal–Wallis tests were used for statistical comparisons.
