# Supplementary material for: Lack of Associations of CHRNA5-A3-B4 Genetic Variants with Smoking Cessation Treatment Outcomes in Caucasian Smokers despite Associations with Baseline Smoking
Source: PLoS One. 2015 May 26;10(5):e0128109. doi: 10.1371/journal.pone.0128109 (PMC4444267; doi:10.1371/journal.pone.0128109)
Supplement: S3 Fig — (DOCX) [file pone.0128109.s003.docx]

**S3 Fig**. The association between *CHRNA5-A3-B4* haplotype rs16969968_rs588765 and smoking abstinence at end of treatment.
