# Supplementary material for: Lack of Associations of CHRNA5-A3-B4 Genetic Variants with Smoking Cessation Treatment Outcomes in Caucasian Smokers despite Associations with Baseline Smoking
Source: PLoS One. 2015 May 26;10(5):e0128109. doi: 10.1371/journal.pone.0128109 (PMC4444267; doi:10.1371/journal.pone.0128109)
Supplement: S1 Table — (DOCX) [file pone.0128109.s004.docx]

**S1 Table**. The two-way interaction table for rs16969968 on smoking cessation.

|  | Odds Ratio | 95% CI | P-value |
| --- | --- | --- | --- |
| Placebo x Nicotine Patch | | | |
| **Genotype Effects:**  **rs16969968_GG_GAAA;** | 0.660 | (0.333 - 1.309) | 0.234 |
| **Treatment Effects:**  **Placebo vs. Nicotine Patch** | 1.036 | (0.508 - 2.115) | 0.922 |
| **Interaction** | 1.524 | (0.602 - 3.854) | 0.374 |
| Placebo x Varenicline | | | |
| **Genotype Effects:**  **rs16969968_GG_GAAA;** | 0.660 | (0.333 - 1.309) | 0.234 |
| **Treatment Effects:**  **Placebo vs. Varenicline** | 1.692 | (0.857 - 3.340) | 0.130 |
| **Interaction** | 1.968 | (0.812 - 4.769) | 0.134 |
| Placebo x Active Treatments | | | |
| **Genotype Effects:**  **rs16969968_GG_GAAA;** | 0.660 | (0.333 - 1.309) | 0.234 |
| **Treatment Effects:**  **Placebo vs. Active Treatments** | 1.342 | (0.728 - 2.473) | 0.345 |
| **Interaction** | 1.743 | (0.783 - 3.878) | 0.174 |
| Nicotine x Varenicline | | | |
| **Genotype Effects:**  **rs16969968_GG_GAAA;** | 1.006 | (0.537 - 1.883) | 0.985 |
| **Treatment Effects:**  **Nicotine vs. Varenicline** | 1.633 | (0.841 - 3.168) | 0.147 |
| **Interaction** | 1.292 | (0.557 - 2.997) | 0.551 |
