# Supplementary material for: Lack of Associations of CHRNA5-A3-B4 Genetic Variants with Smoking Cessation Treatment Outcomes in Caucasian Smokers despite Associations with Baseline Smoking
Source: PLoS One. 2015 May 26;10(5):e0128109. doi: 10.1371/journal.pone.0128109 (PMC4444267; doi:10.1371/journal.pone.0128109)
Supplement: S2 Table — (DOCX) [file pone.0128109.s005.docx]

**S2 Table**. The two-way interaction table for rs588765 on smoking cessation.

|  | Odds Ratio | 95% CI | P-value |
| --- | --- | --- | --- |
| Placebo x Nicotine Patch | | | |
| **Genotype Effects:**  **rs578776_GG_GAAA** | 0.956 | (0.460 - 1.987) | 0.904 |
| **Treatment Effects:**  **Placebo vs. Nicotine Patch** | 1.453 | (0.655 - 3.222) | 0.358 |
| **Interaction** | 0.875 | (0.331 - 2.309) | 0.787 |
| Placebo x Varenicline | | | |
| **Genotype Effects:**  **rs578776_GG_GAAA** | 0.956 | (0.460 - 1.987) | 0.904 |
| **Treatment Effects:**  **Placebo vs. Varenicline** | 2.985 | (1.409 - 6.326) | **0.00432** |
| **Interaction** | 0.772 | (0.307 - 1.941) | 0.583 |
| Placebo x Active Treatments | | | |
| **Genotype Effects:**  **rs578776_GG_GAAA** | 0.956 | (0.460 - 1.987) | 0.904 |
| **Treatment Effects:**  **Placebo vs. Active Treatments** | 2.158 | (1.080 - 4.311) | **0.0294** |
| **Interaction** | 0.8 | (0.345 - 1.856) | 0.604 |
| Nicotine x Varenicline | | | |
| **Genotype Effects:**  **rs578776_GG_GAAA** | 0.836 | (0.442 - 1.583) | 0.583 |
| **Treatment Effects:**  **Nicotine vs. Varenicline** | 2.054 | (1.045 - 4.037) | **0.0367** |
| **Interaction** | 0.883 | (0.378 - 2.063) | 0.774 |
