# Supplementary material for: Lack of Associations of CHRNA5-A3-B4 Genetic Variants with Smoking Cessation Treatment Outcomes in Caucasian Smokers despite Associations with Baseline Smoking
Source: PLoS One. 2015 May 26;10(5):e0128109. doi: 10.1371/journal.pone.0128109 (PMC4444267; doi:10.1371/journal.pone.0128109)
Supplement: S3 Table — (DOCX) [file pone.0128109.s006.docx]

**S3 Table**. The two way interaction table for rs578776 on smoking cessation.

|  | Odds Ratio | 95% CI | P-value |
| --- | --- | --- | --- |
| Placebo x Nicotine Patch | | | |
| **Genotype Effects:**  **rs578776_GG_GAAA** | 1.021 | (0.516 - 2.020) | 0.953 |
| **Treatment Effects:**  **Placebo vs. Nicotine Patch** | 1.537 | (0.841 - 2.812) | 0.163 |
| **Interaction** | 0.730 | (0.291 - 1.830) | 0.502 |
| Placebo x Varenicline | | | |
| **Genotype Effects:**  **rs578776_GG_GAAA** | 1.021 | (0.516 - 2.020) | 0.953 |
| **Treatment Effects:**  **Placebo vs. Varenicline** | 2.364 | (1.323 - 4.225) | **0.00368** |
| **Interaction** | 1.176 | (0.491 - 2.816) | 0.716 |
| Placebo x Active Treatments | | | |
| **Genotype Effects:**  **rs578776_GG_GAAA** | 1.021 | (0.516 - 2.020) | 0.953 |
| **Treatment Effects:**  **Placebo vs. Active Treatments** | 1.928 | (1.142 - 3.252) | **0.0139** |
| **Interaction** | 0.942 | (0.427 - 2.080) | 0.882 |
| Nicotine x Varenicline | | | |
| **Genotype Effects:**  **rs578776_GG_GAAA** | 0.745 | (0.403 - 1.379) | 0.349 |
| **Treatment Effects:**  **Nicotine vs. Varenicline** | 1.538 | (0.887 - 2.667) | 0.125 |
| **Interaction** | 1.611 | (0.708 - 3.665) | 0.256 |
