# Supplementary material for: Lack of Associations of CHRNA5-A3-B4 Genetic Variants with Smoking Cessation Treatment Outcomes in Caucasian Smokers despite Associations with Baseline Smoking
Source: PLoS One. 2015 May 26;10(5):e0128109. doi: 10.1371/journal.pone.0128109 (PMC4444267; doi:10.1371/journal.pone.0128109)
Supplement: S4 Table — (DOCX) [file pone.0128109.s007.docx]

**S4 Table.** The association between *CHRNA5-A3-B4* variants and 6 month and 12 month smoking abstinence in the intent to treat population (N=654)

|  | Placebo | Patch | Varenicline |
| --- | --- | --- | --- |
| **Association with 6 month cessation** | | | |
| RS16969968 | OR=0.537  P=0.132 | OR=0.695  P=0.313 | OR=1.04  P=0.906 |
| RS588765 | OR=0.958  P=0.921 | OR=0.834  P=0.628 | OR=0.849  P=0.622 |
| RS578776 | OR=1.56  P=0.267 | OR=0.751  P=0.427 | OR=0.863  P=0.654 |
| **Association with 12 month cessation** | | | |
| RS16969968 | OR=0.592  P=0.227 | OR=0.78  P=0.502 | OR=0.88  P=0.722 |
| RS588765 | OR=0.869  P=0.755 | OR=1.976  P=0.114 | OR=0.802  P=0.536 |
| RS578776 | OR=1.330  P=0.501 | OR=0.791  P=0.524 | OR=0.892  P=0.747 |

All models were adjusted for age, gender and nicotine metabolism.
