# Supplementary material for: Lack of Associations of CHRNA5-A3-B4 Genetic Variants with Smoking Cessation Treatment Outcomes in Caucasian Smokers despite Associations with Baseline Smoking
Source: PLoS One. 2015 May 26;10(5):e0128109. doi: 10.1371/journal.pone.0128109 (PMC4444267; doi:10.1371/journal.pone.0128109)
Supplement: S5 Table — (DOCX) [file pone.0128109.s008.docx]

**S5 Table.** The association between *CHRNA5-A3-B4* Haplotype and 6 month smoking abstinence in the intent to treat population (N=654).

|  | Effect on Abstinence at 6month | | |
| --- | --- | --- | --- |
| **PREDICTORS** | **Odds Ratio** | **95% CI** | **P** |
| Haplotype |  |  |  |
| Haplotype 1 (G_C) | Reference | | |
| Haplotype 2 (G_T) | 0.78 | 0.39, 1.6 | 0.501 |
| Haplotype 3 (A_C) | 0.57 | 0.24, 1.3 | 0.200 |
| Treatment |  |  |  |
| Placebo | Reference | | |
| Active Treatment | 1.05 | 0.29, 3.86 | 0.937 |
| Interaction of haplotype and treatment |  |  |  |
| Haplotype 1 and active treatment | Reference | | |
| Haplotype 2 and active treatment | 1.12 | 0.49, 2.59 | 0.784 |
| Haplotype 3 and active treatment | 1.62 | 0.62, 4.22 | 0.327 |

All models were adjusted for age, gender and nicotine metabolism.
