# Supplementary material for: Lack of Associations of CHRNA5-A3-B4 Genetic Variants with Smoking Cessation Treatment Outcomes in Caucasian Smokers despite Associations with Baseline Smoking
Source: PLoS One. 2015 May 26;10(5):e0128109. doi: 10.1371/journal.pone.0128109 (PMC4444267; doi:10.1371/journal.pone.0128109)
Supplement: S6 Table — (DOCX) [file pone.0128109.s009.docx]

**S6 Table.** The association between *CHRNA5-A3-B4* Haplotype and 12 month smoking abstinence in the intent to treat population (N=654).

|  | Effect on Abstinence at 12 month | | |
| --- | --- | --- | --- |
| **PREDICTORS** | **Odds Ratio** | **95% CI** | **P** |
| Haplotype |  |  |  |
| Haplotype 1 (G_C) | Reference | | |
| Haplotype 2 (G_T) | 0.76 | 0.36, 1.6 | 0.465 |
| Haplotype 3 (A_C) | 0.61 | 0.25, 1.5 | 0.275 |
| Treatment |  |  |  |
| Placebo | Reference | | |
| Active Treatment | 0.98 | 0.25, 3.85 | 0.973 |
| Interaction of haplotype and treatment |  |  |  |
| Haplotype 1 and active treatment | Reference | | |
| Haplotype 2 and active treatment | 1.30 | 0.54, 3.16 | 0.551 |
| Haplotype 3 and active treatment | 1.40 | 0.51, 3.84 | 0.518 |

All models were adjusted for age, gender and nicotine metabolism.
